# Diversity and Functional Analysis of the FeMo-Cofactor Maturase NifB

**DOI:** 10.3389/fpls.2017.01947

**Published:** 2017-11-14

**Authors:** Simon Arragain, Emilio Jiménez-Vicente, Alessandro A. Scandurra, Stefan Burén, Luis M. Rubio, Carlos Echavarri-Erasun

**Affiliations:** Centro de Biotecnología y Genómica de Plantas, Universidad Politécnica de Madrid (UPM), Instituto Nacional de Investigación y Tecnología Agraria y Alimentaria (INIA), Madrid, Spain

**Keywords:** nitrogenase, iron-molybdenum cofactor, SAM-radical, nitrogen fixation, Azotobacter, methanogens

## Abstract

One of the main hurdles to engineer nitrogenase in a non-diazotrophic host is achieving NifB activity. NifB is an extremely unstable and oxygen sensitive protein that catalyzes a low-potential SAM-radical dependent reaction. The product of NifB activity is called NifB-co, a complex [8Fe-9S-C] cluster that serves as obligate intermediate in the biosyntheses of the active-site cofactors of all known nitrogenases. Here we study the diversity and phylogeny of naturally occurring NifB proteins, their protein architecture and the functions of the distinct NifB domains in order to understand what defines a catalytically active NifB. Focus is on NifB from the thermophile *Chlorobium tepidum* (two-domain architecture), the hyperthermophile *Methanocaldococcus infernus* (single-domain architecture) and the mesophile *Klebsiella oxytoca* (two-domain architecture), showing *in silico* characterization of their nitrogen fixation (*nif*) gene clusters, conserved NifB motifs, and functionality. *C. tepidum* and *M. infernus* NifB were able to complement an *Azotobacter vinelandii* (Δ*nifB*) mutant restoring the Nif^+^ phenotype and thus demonstrating their functionality *in vivo*. In addition, purified *C. tepidum* NifB exhibited activity in the *in vitro* NifB-dependent nitrogenase reconstitution assay. Intriguingly, changing the two-domain *K. oxytoca* NifB to single-domain by removal of the C-terminal NifX-like extension resulted in higher *in vivo* nitrogenase activity, demonstrating that this domain is not required for nitrogen fixation in mesophiles.

## Introduction

Although nitrogen is abundant on Earth, most of it is in the form of dinitrogen (N≡N or N_2_). Due to the strength of its triple bound, N_2_ shows very little reactivity and is therefore not easily available to living organisms (Hoffman et al., 2014). N_2_ fixing organisms (diazotrophs) capable of converting N_2_ into NH_3_, an accessible form of nitrogen, probably appeared in the primordial Earth when the levels of combined nitrogen gradually depleted (Raymond et al., 2004; Canfield et al., 2010). Although evolution and fine-tuning of biological nitrogen fixation (BNF) had an immense impact on the Earth’s nitrogen cycle and allowed life to prosper, only a few bacteria and archaea are actually capable of performing it (Boyd and Peters, 2013). The enzymes that catalyze N_2_ fixation are called nitrogenases (Burris and Roberts, 1993). Nitrogenases are two-component protein complexes, with a catalytic Component I and a Component II acting as obligate electron donor (Bulen and Lecomte, 1966). Three genetically and biochemically distinct classes of nitrogenases have been described to date: the molybdenum nitrogenase, the vanadium nitrogenase, and the iron-only nitrogenase (Bishop and Joerger, 1990). All diazotrophs carry the Mo-nitrogenase and may or may not carry the V or Fe-only ones, referred to as alternative nitrogenases (Dos Santos et al., 2012; Mcglynn et al., 2013). In the case of the Mo-nitrogenase, the Component I is called MoFe protein and is a heterotetramer of the *nifD* and *nifK* gene products, whereas the Component II is called Fe protein and is a homodimer of the *nifH* gene product. A functional nitrogenase complex requires three metal cofactors embedded in the polypeptide chains to reduce N_2_ to NH_3_ (Peters et al., 2011). The NifH homodimer carries a [4Fe–4S] cluster located between the two NifH subunits (Georgiadis et al., 1992), while NifDK harbors an [8Fe–7S] P-cluster at the interface of each NifD (α) and NifK (β) subunits, and an iron-molybdenum cofactor (FeMo-co; [7Fe-9S-C-Mo-homocitrate]) embedded 10 Å beneath the surface of each NifD subunit (Einsle et al., 2002; Spatzal et al., 2011). Alternative nitrogenases contain a third type of subunit in Component I, encoded by *vnfG* (V-nitrogenase) or *anfG* (Fe-only nitrogenase), and either FeV or FeFe cofactors at the active site. These cofactors are proposed to be identical to FeMo-co except for containing V or Fe in place of Mo (Eady, 1996).

NifB stands out as the only protein essential for the activity all nitrogenases (in addition to homocitrate synthase) (Joerger and Bishop, 1988; Dos Santos et al., 2012). NifB is an *S*-adenosyl methionine (SAM)-radical protein that converts [4Fe-4S] clusters into NifB-co, an [8Fe-9S-C] cluster that serves as precursor to FeMo-co, FeV-co and FeFe-co, thus catalyzing the first committed step in nitrogenase active-site cofactor biosynthesis (Shah et al., 1994; Allen et al., 1995; Curatti et al., 2006; George et al., 2008; Wiig et al., 2012) (Supplementary Figure 1). In contrast to FeMo-co, NifB-co is a diamagnetic cluster containing two spectroscopically distinct Fe sites (Guo et al., 2016).

NifB proteins were first purified from the model bacteria *Azotobacter vinelandii* (Curatti et al., 2006) and *Klebsiella oxytoca* (Zhao et al., 2007). The NifB*_Av_* and the NifB*_Ko_* proteins contain a C-terminal NifX-like extension that appears to result from gene fusions during evolution of *nifB* (Boyd et al., 2011). The NifX protein is known to bind and transfer NifB-co to the NifEN scaffold protein for further processing into FeMo-co (Hernandez et al., 2007), but the role of the NifX-domain of NifB is not known. NifB from the archaea *Methanocaldococcus infernus*, expressed and purified from recombinant *Escherichia coli* cells, was stable and enabled biochemical characterization (Echavarri-Erasun et al., 2014). Electron paramagnetic resonance (EPR) studies identified three [4Fe–4S] clusters in NifB*_Mi_*: the SAM-binding [4Fe–4S] cluster and two auxiliary [4Fe–4S] clusters thought to act as substrates for NifB-co synthesis (Wilcoxen et al., 2016). Amino acid residues involved in the coordination of two of these metal clusters were identified by site-directed mutagenesis. NifB*_Mi_* was found capable of FeMo-co synthesis *in vitro*, and exhibited both SAM radical chemistry and SAM demethylation reactions. Additionally, NifB proteins from the archaea *Methanosarcina acetivorans* and *Methanobacterium thermoautotrophicum* purified from recombinant *E. coli* cells were found to catalyze carbide insertion into the FeMo-co precursor (Fay et al., 2015). Importantly, none of the studied archaeal NifB proteins contained the NifX-like extension, showing its dispensability in the *in vitro* FeMo-co synthesis assays for this particular NifB subfamily.

In this work, we have compared the diversity, phylogeny, and domain architecture of 390 putative NifB proteins to understand the minimal requirements for NifB activity. We further used genetic complementation to investigate the *in vivo* functionality of NifB from a hyperthermophilic anaerobic Euryarchaea, a thermophilic anaerobic green sulfur bacterium, and a mesophilic γ-proteobacterium, representing the three existing NifB protein architectures. Finally, NifB from *Chlorobium tepidum* was purified from a recombinant *A. vinelandii* strain and characterized *in vitro*.

## Results

### Generation of a Representative NifB Database

**FIGURE 1:**
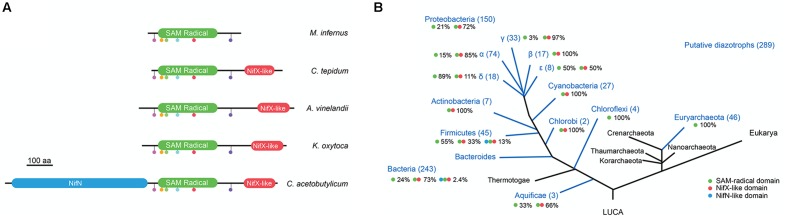
Occurrence NifB architectures in diazotrophs. **(A)** Scheme of the three different NifB architectures and representative species known to possess them. The SAM-radical is shown in green, the NifX-like domain in red, and the NifN-like domain in blue. Color dots represent motifs strictly conserved in the SAM-radical domain. The scale bar represents 100 amino acids. **(B)** Overall distribution and relative frequency of NifB architectures in putative diazotrophs. Green dot represents the SAM-radical domain; red dot represents the NifX-like domain; blue dot represents the NifN-like domain. This panel was generated by overlapping data from Supplementary Table 1 with a 3-domain taxonomic tree of life modified from Boyd and Peters (2013)..

The 390 putative NifB sequences found in the Structure and Function Linkage Database (SFLD) (Akiva et al., 2014) are shown in the Supplementary Table 1. Since SFLD “relates specific sequence-structure features to specific chemical capabilities,” and is therefore not immune to faulty annotations, we identified specific NifB fingerprint motifs and applied them as filter to curate the database. By aligning experimentally proven NifB proteins from *A. vinelandii* (NifB*_Av_*) (Curatti et al., 2006), *K. oxytoca* (NifB*_Ko_*) (Zhao et al., 2007), *Clostridium acetobutylicum* (NifB*_Ca_*) (Chen et al., 2001; Wiig et al., 2011), *M. infernus* (NifB*_Mi_*) (Wilcoxen et al., 2016), *Methanosarcina acetivorans* (NifB*_Ma_*) (Fay et al., 2015), *Methanobacterium thermoautotrophicum* (NifB*_Mt_*) (Fay et al., 2015), and *C. tepidum* (NifB*_Ct_*, this work), a number of conserved motifs were identified in the SAM-radical domain including an HPC motif, the AdoMet motif (Cx_3_Cx_2_C) common to all SAM-radical proteins, an ExRP motif, an AGPG motif, a TxTxN motif and a Cx_2_CRxDAxG motif. Putative NifB proteins that did not present all these motifs were eliminated from the dataset, which was then reduced by 28% down to 289 sequences (**Figure 1** and Supplementary Table 1).

### Phylogenetic Distribution of Three Distinct NifB Domain Architectures

The most widely occurring NifB domain architecture consists of an N-terminal SAM-radical domain linked to a C-terminal NifX-like domain (**Figure 1A**). This protein configuration accounted for 73% of NifB sequences in the Bacteria domain of the curated database (**Figure 1B** and Supplementary Table 2). This configuration has been proposed to emerge after an ancestral gene fusion event (Curatti et al., 2006; Boyd et al., 2011). The functionality of this NifB subfamily has been demonstrated *in vivo* in many bacteria, and *in vitro* for NifB*_Av_* and NifB*_Ko_* (Curatti et al., 2006; Zhao et al., 2007). A second NifB subfamily that included an additional NifN-like domain was found in 6 NifB sequences in the Bacteria domain (corresponding to 2.4% of the curated database). This NifB subfamily was first described in Clostridia (Chen et al., 2001) and then proven functional *in vitro* using purified preparations of an *A. vinelandii* engineered NifN-B fusion that mimicked the Clostridium protein (Wiig et al., 2011). However, *in vivo* complementation of an *A. vinelandii* Δ*nifB* mutant was not shown. Finally, a stand-alone SAM-radical domain was found in 104 NifB sequences, accounting for 100% of the Euryarchaeota and 24% of the Bacteria NifB proteins (**Figure 1B**). The functionality of this NifB subfamily has been demonstrated exclusively *in vitro* for *M. infernus* (NifB*_Mi_*) (Wilcoxen et al., 2016), *M. acetivorans* (NifB*_Ma_*) and *M. thermoautotrophicum* (NifB*_Mt_*) (Fay et al., 2015).

Importantly, the *Clostridium* genus of the Firmicutes phylum is unique in that it contains all three NifB architectures. The curated NifB database contains 45 Firmicutes species likely to be diazotrophic organisms. Among these, 55% carry the stand-alone SAM-radical domain, 33% carry the two-domain architecture, and 13% carry the three-domain architecture (**Figure 1B**).

### NifB Phylogeny Provides Information about the Evolution of Diazotrophs

**FIGURE 2:**
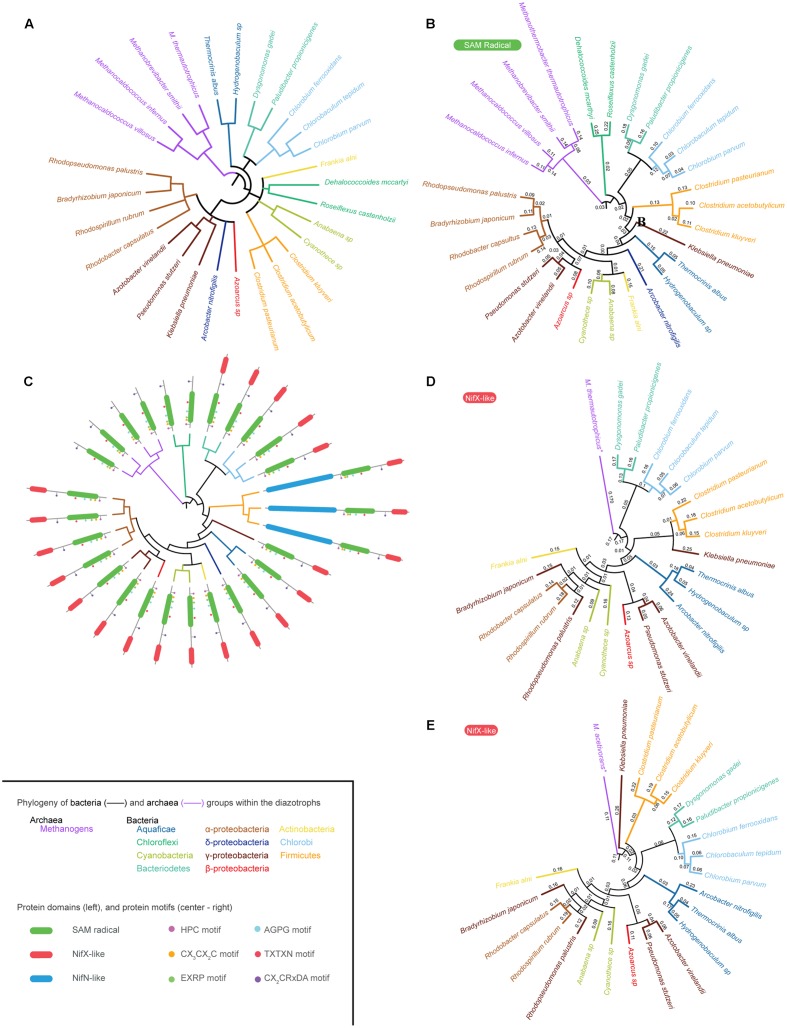
NifB phylogeny. **(A)** Phylogenetic tree of twenty-eight selected species representing all Bacteria and Archaea groups reported to carry *nif* genes. **(B)** Phylogenetic tree of twenty-eight NifB proteins based on their SAM-radical domain. **(C)** Overlap of the phylogenetic tree shown in **(B)** and existing NifB architectures. **(D)** Phylogenetic tree of twenty-two NifB proteins based on their NifX-like domain generated using *M. thermautotrophicus* NifX as root. **(E)** Phylogenetic tree of twenty-two NifB proteins based on their NifX-like domain generated using *M. acetivorans* NifX as root. The inset provides color code for the different Archaea and Bacteria groups with diazotroph members shown in the phylogenetic trees. It also details NifB protein domains and the strictly conserved motifs within the SAM-radical domain **(C)**.

Using the curated NifB database, 28 organisms representing the diversity of phylogenetic groups having diazotrophic members (Boyd and Peters, 2013) were selected to construct a circular phylogenetic tree (**Figure 2A** and Supplementary Table 2) and used as a reference to further overlap NifB phylogenetic trees. In this phylogenetic tree Archaea clade together, as out-group to Bacteria, forming two different subclades: the Methanococci (*M. infernus* and *M. villosus*) and the Methanobacteria (*Methanobrevibacter smithii* and *Methanothermobacter thermautotrophicus*). Bacteria diazotrophic species were distributed as follows: Aquaficae (*Thermocrinis albus* and *Hydrogenobaculum* sp.); Bacteroidetes (*Dysgonomonas gadei* and *Paludibacter propionigenes*), which clade with Chorobi (*Chlorobium ferrooxidans*, *Chlorobium parvum*, and *Chlorobaculum tepidum*); Actinobacteria (*Frankia alni*); Chloroflexi (*Dehalococcoides mccartyi* and *Roseiflexus castenholzii*); Cyanobacteria (*Anabaena* sp. and *Cyanothece* sp.); and Firmicutes (*Clostridium kluyveri*, *C. acetobutylicum*, and *C. pasteurianum*), all found in the same clade. Finally, α-proteobacteria (*Rhodopseudomonas palustris*, *Bradyrhizobium japonicum*, *Rhodospirillum rubrum*, and *Rhodobacter capsulatus*), β-proteobacteria (*Azoarcus* sp.), γ-proteobacteria (*A. vinelandii*, *K. oxytoca* and *Pseudomonas stutzeri*), and δ-proteobacteria (*Arcobacter nitrofigilis*) were all in the same clade.

Because of the existence of three different NifB architectures, poorly aligned segments could potentially distort the phylogenetic tree analyses. Therefore, these regions were removed with Gblocks software (Talavera and Castresana, 2007) leaving a 315 contiguous amino acid sequence that was used to generate the SAM-radical domain tree (**Figures 2B,C**) and a 64 contiguous amino acid sequence used to generate two different NifX-like domain trees (**Figures 2D,E**).

The SAM-radical domain tree was rooted in *M. infernus* and is shown in **Figure 2B**. A derivative tree illustrating the distribution of NifB domain architecture is presented in **Figure 2C**. The Aquaficae, Actinobacteria, and Cyanobacteria did not clade with Firmicutes, as expected according to **Figure 2A**, but with Proteobacteria classes, leaving the Firmicutes as out-group to all of them. Interestingly, the γ-proteobacteria NifB*_Ko_* was found as out-group to all proteobacteria in agreement with previous analysis (Boyd et al., 2011). The Chlorobi and Bacteroidetes NifB claded as expected. However, Chloroflexi NifB rooted deeper in the Bacteria, being the closest relative to Archaea NifB. Previous studies proposed that the entire *nif* operon might have been laterally transferred to Chloroflexi from an ancestral methanogen co-existing in a common ecological niche (Eisen et al., 2002). Our data support this hypothesis.

The phylogenetic signal of the NifX-like domain of NifB was also analyzed (**Figures 2D,E**). No Archaea NifB with a NifX-like domain has to our knowledge been found. Chloroflexi also lack this domain, suggesting acquisition from Archaea by a lateral gene transfer event (LGT) as previously suggested (Eisen et al., 2002; Boyd et al., 2011). Distinct NifX proteins encoded in the genomes of some methanogens were then used to root the trees. Two substantially different phylogenetic trees were obtained depending on the NifX protein used as root. Since NifX was not found in any Methanococci (i.e., *M. infernus*), **Figure 2D** uses NifX from Methanobacteriales (*M. thermautotrophicus*) and **Figure 2E** uses NifX from Methanosarcinales (*M. acetivorans*). The pattern of the first tree is similar to that of the SAM-radical domain tree, suggesting Chloroflexi as the bacterial ancestor from which the lineage emerged. The second tree, however, points to Firmicutes as the bacterial ancestor from which *nif* genes proliferated in Bacteria. This remains an interesting possibility given that Firmicutes present the three different NifB architectures known to date.

### Organization of *nif* Genes in the Genomes of *C. tepidum and M. infernus*

In order to define essential and not essential domains for NifB function *in vivo* in an aerobic mesophilic host, we focused on NifB from the thermophile *C. tepidum* (two-domain architecture), the hyperthermophile *M. infernus* (single-domain architecture), and the mesophile *K. oxytoca* (two-domain architecture). NifB*_Ko_* and NifB*_Mi_* have previously been purified and characterized *in vitro* (Zhao et al., 2007; Wilcoxen et al., 2016) but not NifB*_Ct_*, which is reported in this study.

*Chlorobium tepidum* is a well-described diazotroph (Wahlund and Madigan, 1993) with annotated genome (Eisen et al., 2002). Most of its *nif* genes are located in a single 20-kb cluster containing the Mo-nitrogenase structural genes (*nifH*, *nifD*, and *nifK*), FeMo-co biosynthetic genes (*nifB*, *nifE, nifN*, *nifV*, and *fdxN*), and regulatory genes (*nifA*, *nifI*_1_, and *nifI*_2_) (Supplementary Figure 2). Genome blast with individual *nif* genes from the model *diazotroph K. oxytoca* did not reveal anomalies, supporting the current *C. tepidum* annotation.

While NifB*_Mi_* expressed in *E. coli* was shown to support FeMo-co synthesis *in vitro* (Wilcoxen et al., 2016), *M. infernus* has not yet been proven to be diazotrophic. The *nif* genes in the *M. infernus* genome consist of *nifH*, *nifD*, and *nifK* structural genes, *nifB* and *nifE* cofactor biosynthetic genes, and *nifI*_1_ and *nifI*_2_ regulatory genes. Intriguingly, a second *nifH* gene is located 17-kb apart from the *nif* cluster and *nifB* was found 470-kb apart with no apparent *nif* genes in close proximity.

### NifB*_Ct_* and NifB*_Mi_* are Functional *in Vivo* When Expressed in the Aerobic Mesophilic Host *A. vinelandii*

**FIGURE 3:**
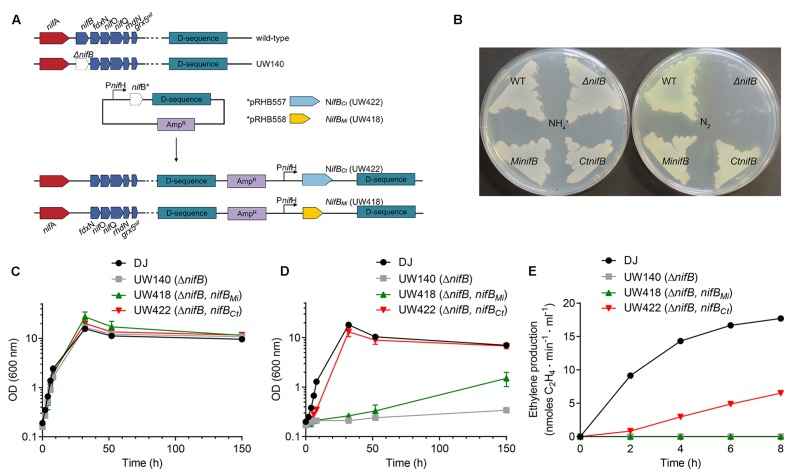
Genetic complementation of *A. vinelandii* Δ*nifB* with *nifB_Ct_* and *nifB_Mi_* genes. **(A)** Scheme showing the construction of *A. vinelandii* Δ*nifB* derivative strains carrying *nifB_Mi_* (UW418) and *nifB_Ct_* UW422 inserted in their chromosome under the control of a copy of the *nifH* promoter. **(B)** Petri dishes with solid Burk media showing growth of *A. vinelandii* DJ (wild-type strain, WT), UW140 (Δ*nifB*), UW418 (Δ*nifB*, P*nifH::nifB_Mi_*) and UW422 (Δ*nifB*, P*nifH::nifB_Ct_*) in the presence (left plate) or absence (right plate) of ammonium as nitrogen source. **(C,D)** Growth curves of DJ, UW140, UW418 and UW422 strains using ammonium **(C)** or N_2_ **(D)** as source of nitrogen. **(E)** *In vivo* acetylene reduction activity of *nif* derepressed *A. vinelandii* strains shown as nmol ethylene formed⋅min^-1^⋅ml^-1^ at a normalized OD of 1. UW140 and UW422 strains did not exhibit detectable activities during the 8-h period following derepression. Data in **(C–E)** represent means ± SD (*n* ≥ 2).

Genetic complementation analyses were performed by expressing synthetic codon-optimized *nifB_Ct_* and *nifB_Mi_* genes in the *A. vinelandii* UW140 (Δ*nifB*) strain under the control of the *nifH* promoter (**Figure 3A**). *A. vinelandii* is a strict aerobe with optimum growth temperature of 30°C and is used here to provide an initial screen of NifB functionality that will be useful for further screening and implementation in Eukaryotic hosts. Strains UW418 (Δ*nifB*, *PnifH*::*nifB_Mi_*) and UW422 (Δ*nifB*, *PnifH*::*nifB_Ct_*) exhibited diazotrophic growth both in solid and liquid culture media (**Figures 3B,D**), in contrast to the Nif^-^ phenotype of the parental strain UW140 (Δ*nifB*). Calculated diazotrophic growth rates (ln2/t*_d_*) were: 0.23 for the wild type, <0.001 for UW140, 0.015 for UW418, and 0.13 for UW422. This data shows that, although both NifB*_Ct_* and NifB*_Mi_* originate from strict anaerobic and thermophilic microbes, the proteins were functional and could complement the *A. vinelandii* Δ*nifB* mutant phenotype. However, whereas NifB*_Ct_* supported similar growth rate at 30°C as the *A. vinelandii* wild type strain, the recombinant NifB*_Mi_* did not, possibly explained by the almost 40°C difference in optimal growth temperature between *C. tepidum* (48°C, Wahlund and Madigan, 1993) and *M. infernus* (85°C, Jeanthon et al., 1998). No difference in growth rate could be observed when using NH_4_^+^ as nitrogen source: 0.31 for the wild type, 0.29 for UW140, 0.30 for UW418, and 0.30 for UW422 (**Figure 3C**).

*In vivo* nitrogenase activities determined by the acetylene reduction assay showed significant activity in UW422 in the 8 h period following nitrogenase derepression (**Figure 3E**). No activity was detected in UW418 within this period of time, consistent with its significantly lower diazotrophic growth rate.

### Purification and Biochemical Characterization of NifB*_Ct_*

**FIGURE 4:**
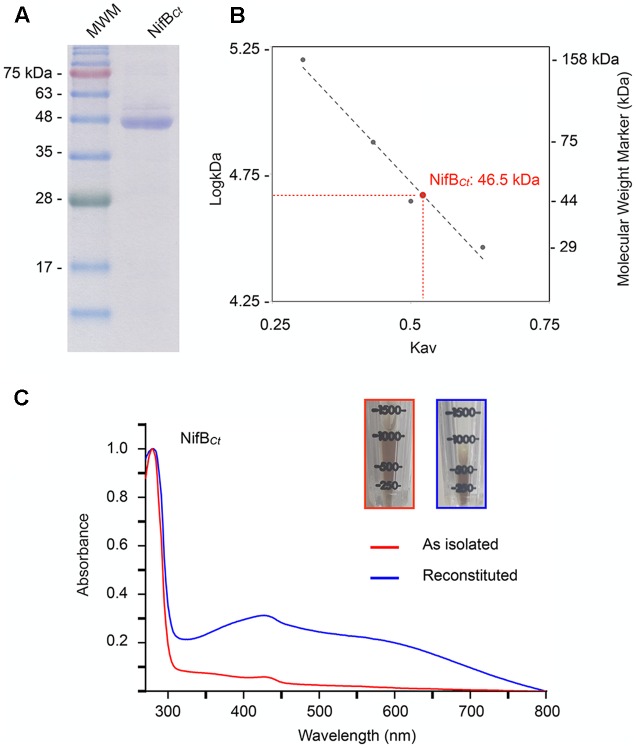
Molecular mass and UV-vis spectra of purified NifB*_Ct_* preparations. **(A)** SDS-PAGE analysis of NifB*_Ct_* purified from recombinant *A. vinelandii* UW422. **(B)** NifB*_Ct_* native molecular weight as determined by size-exclusion chromatography against known protein weight markers. **(C)** UV-vis spectra of the as isolated (red) and [Fe–S] cluster reconstituted (blue) preparations of pure NifB*_Ct_*. The inset pictures show the increase in color intensity upon [Fe–S] cluster reconstitution.

NifB*_Ct_* was expressed and purified from a recombinant *A. vinelandii* strain (**Figure 4A**). The yield of pure NifB*_Ct_* from *A. vinelandii* cells was 0.3 μg NifB*_Ct_* per gram of cell, 15-fold higher than that of overexpressed NifB*_Av_* (Curatti et al., 2006). Purity of the NifB*_Ct_* preparations exceeded 95%, as determined by Coomassie stained SDS-gels, and the identity of NifB*_Ct_* was confirmed by MALDI-TOF analysis with 60% sequence coverage (Supplementary Table 3). NifB*_Ct_* migrated as a monomer of 46.5 kDa in anaerobic size exclusion chromatography (**Figure 4B**), in good agreement with theoretical mass determined by the amino acid sequence (46.8 kDa). As isolated NifB*_Ct_* contained 3.05 Fe atoms per monomer. *In vitro* reconstitution of its [Fe–S] clusters under reducing conditions increased Fe contents to 10.1 ± 0.07 Fe atoms (*n* = 3). Consistently, features characteristic of [Fe–S] proteins (especially the broad shoulder at 400-420 nm) were more prominent in the reconstituted NifB*_Ct_* UV-vis spectrum (**Figure 4C**). Reconstituted NifB*_Ct_* was active in the *in vitro* FeMo-co synthesis and nitrogenase activation assay: 5.2 ± 2.2 nmol ethylene formed⋅min^-1^⋅assay^-1^ (*n* = 2) compared to 8.2 ± 1.5 nmol ethylene formed⋅min^-1^⋅assay^-1^ (*n* = 2) when using pure NifB-co.

### The NifX-like Domain of NifB*_Ko_* Is Not Essential for Nitrogenase Activity or Diazotrophic Growth

**FIGURE 5:**
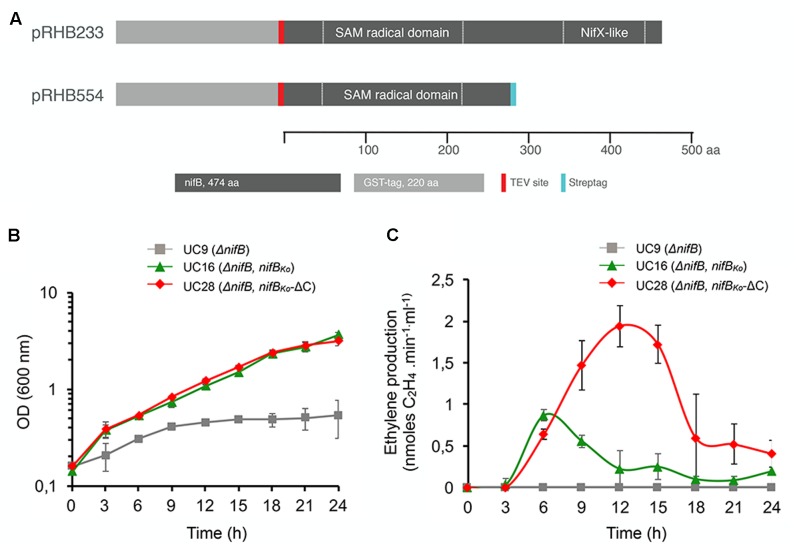
**(A)** Scheme of full-length GST-NifB*_Ko_* and GST-NifB*_Ko_*-ΔC in expression plasmids carried by UC16 and UC28, respectively. **(B,C)** Diazotrophic growth **(B)** and *in vivo* acetylene reduction activity **(C)** of *K. oxytoca* UC16 and UC28 expressing full-length and truncated NifB variants, respectively, compared to the UC9 (Δ*nifB*) strain. Data represent means ± SD (*n* = 3). Maximum ethylene production of *K. oxytoca* UN (wild-type) was 12.6 nmol⋅min^-1^⋅ml^-1^ (*n* = 3).

The capacity of *nifB_Mi_* to complement the Δ*nifB* strain strongly suggests that the SAM-radical domain of NifB is the only one required for the synthesis of the FeMo-co precursor, but this could be a property specific to the stand-alone SAM-radical domain subfamily. To determine whether the NifX-like domain naturally present in the two-domain NifB architecture is required for NifB-co synthesis, a truncated NifB*_Ko_* variant lacking the entire NifX-like domain (*nifB_Ko_-*ΔC) was generated, introduced in *K. oxytoca* UC9 (Δ*nifB*) and expressed under the control of a *tac* promoter (**Figure 5A** and Supplementary Figure 3). Additionally, as this truncated version would mimic a mesophilic single-domain NifB, we could test whether presence of the NifX-like domain is important for growth under moderate, non-thermophilic, temperatures. Diazotrophic growth and *in vivo* nitrogenase activity of UC28 (Δ*nifB*, *Ptac*::*nifB_Ko_-*ΔC) were measured at 3 h intervals in a 24 h time course following derepression and compared to those of UC16 (Δ*nifB*, *Ptac*::*nifB_Ko_*), a control strain expressing full-length NifB*_Ko_*. Surprisingly, UC28 exhibited diazotrophic growth similar to UC16 and *in vivo* nitrogenase activity higher than UC16 (**Figures 5B,C**). The UC9 parental strain did not exhibit nitrogenase activity or diazotrophic growth, confirming that the functionality of the expressed NifB*_Ko_* variants and suggesting that the NifX-like extension of NifB*_Ko_* is not required for NifB-co synthesis, at least under the growth conditions tested in this study, and that this could be a general rule for the two-domain family of NifB proteins.

## Discussion

### NifB Phylogeny and Architecture

To our knowledge, this work presents the largest compilation of NifB proteins described to date. The NifB database was stringently filtered to exclude faulty annotated proteins and the curated dataset provides insights about NifB origin, taxonomy and architecture that complement previous work (Soboh et al., 2010; Boyd et al., 2011; Boyd and Peters, 2013). In this study we demonstrate that the SAM-radical domain of NifB is sufficient to support FeMo-co biosynthesis *in vivo* in the model organisms *A. vinelandii* and *K. oxytoca*.

A strict filter, based on motifs exhibited by experimentally confirmed NifB proteins, was applied to the initial database. As a result, 28% NifB sequences were excluded from further analysis. Although these criteria might be too strict, we reasoned that it was better to miss some true-positives than to risk including false-positives. Most excluded NifB proteins lacked the conserved Cx_3_Cx_2_C motif required for SAM-radical catalysis. In contrast, the NifX domain was identified in each one of them and we think that these faulty annotated NifB proteins are instead NifX. This confusion originates from the fact that the NifX domain is present in NifB, NafY, NifY as well as NifX proteins.

Three distinct NifB protein architectures exist. The most widespread in Bacteria consists of an N-terminal SAM-radical domain followed by a C-terminal NifX-like domain. However, this configuration is absent in Archaea, which present smaller NifB proteins consisting of a stand-alone SAM-radical domain. Boyd and collaborators investigated the lineage of the stand-alone SAM-radical domain in Archaea NifB proteins and compared it to the two-domain architecture favored in Bacteria (Boyd et al., 2011). The authors traced an event that suggested that a methanogen donated its *nif* cluster via LGT to a Firmicutes ancestor that co-existed in the same ecological niche. Then, a fusion event happened that resulted in the *nifB-nifX* protein occurring in Firmicutes. It was later suggested that the wide spread of the *nifB-nifX* fusion protein in Bacteria was independent of the selective pressure associated with aerobic diazotrophy (Boyd et al., 2015). An additional fusion event between *nifN* and *nifB-nifX* also occurred in Firmicutes leading to the three-domain NifB architecture. This last event was confined to Firmicutes, which is the only phylum presenting all three types of NifB architecture. It is surprising that the three-domain NifB was not widespread in Bacteria. From knowledge gained through *in vitro* FeMo-co synthesis studies (Curatti et al., 2007), it could be assumed that a NifENB fusion protein would be beneficial by protecting labile NifB-co and streamlining FeMo-co synthesis. However, it is possible that a NifENB fusion might not allow fine-tuning of precursor biosynthesis.

Based on the phylogeny of independent NifX proteins, another early *nifB* LGT was detected between Methanosarcinales and Chloroflexi. This event was also apparent in the SAM-radical domain phylogenetic tree, with Chloroflexi rooting deeper than any other group. The short distance between Methanosarcinales and Chloroflexi NifB lineages was also observed by Boyd and colleagues (Boyd et al., 2011).

### Ancestral NifB Proteins from Strict Anaerobic and Thermophilic Organisms that Function *in Vivo* in an Aerobic Mesophilic Host

Stand-alone SAM-radical domain NifB proteins catalyze NifB-co synthesis *in vitro* (Fay et al., 2015; Wilcoxen et al., 2016). However, they have not yet been proven capable of sustaining diazotrophic growth of *M. thermautotrophicus*, *M. acetivorans*, and *M. infernus* (which also are not yet experimentally confirmed to be diazotrophs). It was also not clear whether this NifB family would function in a mesophilic and aerobic environment, which could prevent their use for plant nitrogenase engineering. Therefore, the Nif^+^ phenotype exhibited by the *A. vinelandii ΔnifB* strain complemented with *nifB_Mi_* presented in this study is convincing evidence of its *in vivo* functionality in a mesophilic and aerobic bacterium.

As expected, stronger Nif^+^ phenotype was achieved by complementation with NifB*_Ct_*. *C. tepidum* is a mild thermophile with optimum growth temperature of 48°C and therefore much closer to the 30°C optimum of *A. vinelandii.* In addition, NifB*_Ct_* has a two-domain NifB architecture similar to NifB*_Av_*. Interestingly, NifB*_Ct_* was a monomer, similar to the archaeal single-domain NifB proteins and different from the NifB*_Av_* and NifB*_Ko_* homodimers. Although constrained by the limited set of available experimental data, it appears that NifB monomers might be more stable and therefore favored in thermophilic organisms regardless of protein architecture. Importantly, both configurations are functional *in vivo* in a mesophilic host. The strong diazotrophic growth of UW418 in plates compared to liquid medium suggests that there are other factors limiting NifB*_Mi_* activity *A. vinelanii* in addition to operational temperature. One possibility is that oxygen limitation during growth in plate has a positive effect on NifB*_Mi_* that is not observed in liquid medium.

### The NifX-like Domain of NifB*_Ko_* May Have a Role Regulating the Flux of NifB-co during FeMo-Co Biosynthesis

It was suggested that the distinct NifB*_Av_* domain architecture (the N-terminal SAM-radical domain and the C-terminal NifX-like domain) could be required to coordinate [Fe–S] cluster precursors prior to catalysis resulting in NifB-co synthesis (Curatti et al., 2006). This possibility was put into question when stand-alone SAM-radical domain archaeal NifB were found active *in vitro* (Arragain et al., 2014; Fay et al., 2015; Wilcoxen et al., 2016). Here, we demonstrate that the NifX-like domain of NifB*_Ko_* is not essential for catalytic activity *in vivo*. A truncated NifB*_Ko_* lacking the NifX-like domain supported *in vivo* nitrogenase (ethylene production) rates even higher than full-length NifB. It is thus reasonable to think that NifB catalysis only requires the SAM-radical domain, and that other domains may perform complementary functions that are beneficial but not essential for FeMo-co biosynthesis. A critical role in cofactor biosynthesis for alternative nitrogenases is not likely as this domain is absent in NifB from *M. acetivorans*, which carries all three types of nitrogenase (Galagan et al., 2002).

### Prospects to Implement NifB Activity in Eukaryotes

The successful purification of active NifH from yeast mitochondria, when co-expressed with NifU, NifS and NifM, represented a first advance toward implementing BNF in eukaryotic systems (Lopez-Torrejon et al., 2016). However, major steps are still required to engineer active nitrogenase in a eukaryote. In this regard, expression of functional NifB is expected to be a major barrier to overcome. This is not only because NifB catalyzes a reaction unique and essential to diazotrophs, but also because of the O_2_-labilility of its [Fe-S] clusters, including NifB-co.

NifB from well-established model organisms, such as *A. vinelandii* and *K. oxytoca*, might be difficult to use in the harsh environment provided by a eukaryotic cell. There is evidence that NifB catalysis makes it susceptible to proteolysis (Martinez-Noel et al., 2011). Screening for simpler, but more suitable variants from less “sophisticated” diazotrophs may be a rewarding strategy. In this aspect, the use of less labile, monomeric, and temperature-resistant NifB from Archaea or Bacteria, such as the two examples shown in this study, may help engineering FeMo-co biosynthesis in Eukaryotic (plant) cells. The accompanying paper (Burén et al., 2017) describes the first successful step in this direction.

## Materials and Methods

### Data Mining and Phylogenetic Analysis

The 390 annotated NifB sequences retrieved from the Structure and Function Linkage Database (SFLD) (Akiva et al., 2014) and UniProt^1^ are shown in Supplementary Table 1. To exclude potentially faulty annotated sequences, the following filtering procedure was applied to the dataset. First, amino acid sequences of experimentally proven NifB proteins, including *A. vinelandii* (NifB*_Av_*) (Curatti et al., 2006), *K. oxytoca* (NifB*_Ko_*) (Zhao et al., 2007), *Clostridium pasteurianum* (NifB*_Cp_*) (Chen et al., 2001; Wiig et al., 2011), *M. infernus* (NifB*_Mi_*) (Wilcoxen et al., 2016), *Methanosarcina acetivorans* (NifB*_Ma_*) (Fay et al., 2015), *Methanobacterium thermoautotrophicum* (NifB*_Mt_*) (Fay et al., 2015), and *C. tepidum* (this work) were aligned to determine conserved motifs. These NifB fingerprint motifs localized in the SAM-radical domain and included an HPC motif, the AdoMet Cx_3_Cx_2_C motif, an ExRP motif, an AGPG motif, a TxTxN motif, and a Cx_2_CRxDAxG motif (**Figure 1**). The full NifB dataset was then analyzed for the presence of these fingerprints, reducing the initial 390 sequences to 289 (Supplementary Table 1). Protein domain architecture was analyzed using the PFAM database^2^ (Finn et al., 2016). The frequency of appearance of each one of the different NifB domains in diazotrophic phyla shown in **Figure 1** was represented by overlapping data from Supplementary Table 1 with a 3-domain taxonomic tree of life (modified from Boyd and Peters, 2013).

Twenty-eight NifB proteins representing all phylogenetic groups known to contain diazotrophs (Boyd and Peters, 2013) (Supplementary Table 2) were selected from the reduced list and used to investigate taxonomy versus architecture correlation. The taxonomy of diazotrophic groups was resolved using PhyloT^3^, an online tool that uses the full NCBI taxonomy to generate phylogenetic trees (**Figure 2A**).

Clustal Omega^4^ was used to generate protein alignments and neighbor joining (NJ) phylogenetic trees (Sievers et al., 2011). Maximum likehood (ML) trees shown in **Figures 2B–E** were produced using the IQ-Tree web server^5^ (Trifinopoulos et al., 2016). Gblocks (Talavera and Castresana, 2007) was used to remove non-conserved aligned segments leaving a 315 contiguous amino acid sequence that was used to generate the SAM-radical domain tree (**Figures 2B,C**) and a 64 contiguous amino acid sequence used to generate the NifX-like domain trees (**Figures 2D,E**). Phylogenetic trees shown in **Figures 2B–E** were resolved using the Interactive Tree of Life online tool^6^ (Letunic and Bork, 2007) and FigTree.

### Plasmids, Strains and Growth Conditions

The strains and plasmids used in this work are listed in Supplementary Table 4. *A. vinelandii* strains DJ (wild-type) (D.R. Dean, Virginia Tech) and UW140 (Δ*nifB*) (Hernandez et al., 2007) have been described. *K. oxytoca* strains UC9 (Δ*nifB*) and UC16 (Δ*nifB*, P*tac*::*gst-nifB_Ko_*) (Zhao et al., 2007) have been described.

The *M. infernus* (*nifB_Mi_*, accession number D5VRM1) and *C. tepidum* (*nifB_Ct_*, accession number. CT1540) *nifB* sequences were codon-optimized and synthesized by GenScript (Piscataway, NJ, United States) for expression in *E. coli*. Plasmids pRHB557 and pRHB558 contained the *nifB_Ct_* and *nifB_Mi_* genes, respectively, cloned into the *Nde*I and *EcoR*I sites of pRHB258 for the expression of His_9_-tagged proteins under the control of the *nifH* promoter (Curatti et al., 2007). Plasmids pRHB557 and pRHB558 were inserted into the chromosome of *A. vinelandii* UW140 (Δ*nifB*) by homologous recombination at the D-sequence, a 1.1-kb DNA fragment from the chromosomal region downstream of Avin02530 (Hernandez et al., 2008), to generate strains UW422 and UW418, respectively (**Figure 3A**). Transformants were selected in agar plates of NH_4_^+^-free Burk’s modified medium (Shah et al., 1972) containing 50 μg/ml ampicillin.

For diazotrophic growth rate *A. vinelandii* strains were grown at 30°C on N-free Burk’s medium. When a fixed nitrogen source was required, ammonium acetate was added to a final concentration of 29 mM. Growth was estimated as OD_600_ using an Ultrospec 3300 Pro spectrophotometer (Amersham). The exponential growth rate constant corresponds to ln2/td, where td represents the doubling time.

For *A. vinelandii in vivo* nitrogenase activity determinations strains were grown at 30°C on NH_4_^+^ supplemented Burk’s medium and then collected, washed and derepressed for nitrogenase as previously described (Shah et al., 1972). Acetylene reduction was determined as described in (Stewart et al., 1967).

Expression plasmid pRHB233 (P*tac*::*gst-nifB_Ko_*) is a derivative of pGEX-4T-3 (GE Healthcare) that contains the entire *nifB_Ko_* gene (1404 nucleotides encoding a 468 amino-acid polypeptide; UniProt accession number P10390) fused to a *gst*-encoding gene (Zhao et al., 2007). Plasmid pRHB233 was used as template to amplify a truncated *nifB_Ko_* variant using oligonucleotides 5′-CCCCATATGACTACTTCCTGCTCCTCTTTTTCTGGCGGC-3′ and 5′-GGGCTCGAGTCAATGATGATGATGATGATGATGATGATGCGCGGGTCGCAATGCTGGCGTGCAG-3′. The resulting 1008 bp fragment, encoding a 336 amino acid NifB*_Ko_* polypeptide that lacked the C-terminal NifX-like domain (NifB*_Ko_-*ΔC), was cloned into the *Nde*I and *Xho*I sites of pGEX-4T-3 to generate plasmid pRHB554. *K. oxytoca* UC9 (Δ*nifB*) strain was transformed with pRHB554 to generate strain UC28 (Δ*nifB*, P*tac*::*gst-nifB_Ko-_ΔC*). Positive transformants were selected in LC agar plates containing ampicillin (150 μg/ml) and carbenicillin (800 μg/ml).

For diazotrophic growth rate and *in vivo* nitrogenase activity determinations, *K. oxytoca* strains were grown overnight at 30°C in minimal medium supplemented with 28.5 μM ammonium acetate (Shah et al., 1994). Cells were washed three times using N-free medium and finally resuspended at a final OD_600_ value of 0.15 in N-free medium supplemented with 0.1% serine, 150 μg/ml ampicillin, 800 μg/ml carbenicillin, and 5 μM IPTG in dual-sealed 100-ml vials under O_2_-free conditions. At 3-h intervals during a period of 24 h, culture growth was monitored by OD_600_ using an Ultrospec 3300 Pro spectrophotometer (Amersham), and the *in vivo* nitrogenase activity was determined by ethylene production at 30°C for 30 min in 1-ml culture samples at a normalized OD_600_ value of 1, as previously described (Stewart et al., 1967). The growth rate constant corresponds to ln2/td, where td represents the doubling time.

### Purification of NifB*_Ct_* from *A. vinelandii* Recombinant Cells

*Azotobacter vinelandii* UW422 cells overexpressing NifB*_Ct_* under the control of a *nifH* promoter were grown in 32-l batches in a 300-l fermenter (Bioprocess Technology). Nitrogenase derepression and cell collection were carried out as described in (Echavarri-Erasun et al., 2014).

Purification of His-NifB*_Ct_* from *A. vinelandii* cells was as follows: 150 g of cells were resuspended in 450 ml buffer A (50 μM Na_2_HPO_4_, pH 7.6, 4 M glycerol, 5 μM 2-mercaptoethanol and 2 μM Na_2_S_2_O_4_) supplemented with protease inhibitors (200 μM PMSF and 1 μg/ml leupeptin) and 5 μg/ml DNAse inside a Coy Labs glovebox for 30 min. Cells were pelleted at 14,000 × *g* for 10 min at 4°C and then transferred back inside the glovebox. Pellets were lysed by osmotic shock in 450 ml buffer B (50 μM Na_2_HPO_4_, pH 7.6, 0.05% *n*-dodecyl-β-D-maltoside, 5 μM 2-mercaptoethanol and 2 μM Na_2_S_2_O_4_). A cell-free extract was obtained by collecting the supernatant after centrifugation at 70,000 × *g* for 1 h at 4°C. The cell-free extract was supplemented with NaCl to a final concentration of 180 μM and loaded onto a 25-ml IMAC column (GE Healthcare) previously charged with Co^2+^ and equilibrated in buffer C (50 μM Na_2_HPO_4_, pH 7.6, 180 μM NaCl, 0.05% n-dodecyl-β-D-maltoside, 5 μM 2-mercaptoethanol, 10% glycerol and 2 μM Na_2_S_2_O_4_) at 4°C. Column was washed with 3 column volumes of buffer C, followed by 7 column volumes of buffer C supplemented with 50 μM imidazole. NifB*_Ct_* was eluted using buffer C supplemented with 300 μM imidazole. Eluted fractions were analyzed by SDS-PAGE and Coomassie staining. Fractions containing pure NifB*_Ct_* were pooled and desalted using a HiPrep 26/10 desalting column (GE Healthcare) previously equilibrated with buffer C. Purified NifB*_Ct_* was stored in liquid N_2_ as pellets.

### Determination of NifB*_Ct_* Native Molecular Weight

NifB*_Ct_* Native Molecular Weight was determined by size-exclusion chromatography using a HiLoad 16/600 Superdex 200 column attached to an AKTA FPLC (GE Healthcare). The column was equilibrated with 50 μM Na_2_HPO_4_, pH 7.6, 180 μM NaCl, 10% glycerol, 5 μM 2-mercaptoethanol and 2 μM Na_2_S_2_O_4_ and the chromatography was run with the same buffer at a flow rate of 1 ml/min. The column was calibrated for molecular mass determination by using the molecular weight standard proteins aldolase (158 kDa), conalbumin (75 kDa), ovalbumin (44 kDa), and carbonic anhydrase (29 kDa) (GE Healthcare).

### NifB*_Ct_* [Fe–S] Cluster Reconstitution

As isolated NifB*_Ct_* samples were diluted in 50 μM Tris-HCl (pH 8) buffer containing 200 mM KCl and 10% glycerol to a final concentration of 10 μM NifB*_Ct_*. Samples were then incubated during 2 h at 37°C with a 12-fold molar excess of Fe^2+^ [(NH_4_)_2_Fe(SO_4_)_2_] and S^2-^ (Na_2_S), in the presence of 10 μM DTT. The Fe and S excess was removed from reconstituted preparations by filtration in a HiPrep 26/10 desalting column (GE Healthcare) equilibrated in dilution buffer. After desalting, Fe content of reconstituted NifB*_Ct_* samples was quantified as described by Fish (1988).

### NifB*_Ct_*-Dependent *in Vitro* Synthesis of FeMo-co

*Azotobacter vinelandii* UW140 (Δ*nifB*) cell-free extracts were obtained as described above and used for biochemical complementation assays. Purified NifB*_Ct_* (0.16 μM) was added to reaction mixtures containing 0.2 ml of UW140 cell-free extract (4.4 μg protein/ml) in 22 μM Tris-HCl (pH 7.4), 17.5 μM Na_2_MoO_4_, 175 μM *R*-homocitrate, 400 μM (NH_4_)_2_FeSO_4_, 400 μM Na_2_S, 880 μM SAM, 1.32 μM ATP, 18 μM phosphocreatine, 2.2 μM MgCl_2_, 3 μM Na_2_S_2_O_4_, 3.5% glycerol, 40 μg creatine phosphokinase, and 2 μM NifH at a final volume of 400 ml. Control reactions contained 1.4 μM pure NifB-co instead of NifB*_Ct_*. Reactions were incubated at 30°C for 45 min inside an MBraun glovebox (O_2_ < 0.1 ppm) to allow for FeMo-co synthesis and insertion into apo-NifDK present in the UW140 extract. Acetylene reduction activity of reconstituted NifDK protein was quantified after addition of 0.1 μg NifH, changing the vial gas phase to 100% argon, and finally injecting 0.5 ml acetylene. Reaction mixtures were incubated in a water bath at 30°C for 15 min and 600 rpm shaking and then stopped by addition of 0.1 ml of 8 M NaOH. Ethylene formation was measured in a Shimadzu GC-2014 gas chromatograph equipped with a Porapak N80/100 column.

## Author Contributions

CE-E, SA, EJ-V, and AS carried out experimental work; CE-E, SA, EJ-V, SB, and LR carried out experimental design and data analysis; CE-E, SB, and LR wrote the paper.

## Conflict of Interest Statement

The authors declare that the research was conducted in the absence of any commercial or financial relationships that could be construed as a potential conflict of interest.

## Footnotes

1 http://uniprot.org

2 http://pfam.xfam.org

3 http://phylot.biobyte.de/

4 http://www.ebi.ac.uk/Tools/msa/clustalo/

5 http://www.iqtree.org

6 http://itol.embl.de
