# Supplementary material for: Diversity and Functional Analysis of the FeMo-Cofactor Maturase NifB
Source: Front Plant Sci. 2017 Nov 14;8:1947. doi: 10.3389/fpls.2017.01947 (PMC5715403; doi:10.3389/fpls.2017.01947)
Supplement: Supplementary file 1 [file Presentation_1.pdf]

Supplementary Material

Diversity and Functional Analysis of the FeMo-cofactor Maturase NifB

Simon Arragain[1,2,§], Emilio Jiménez-Vicente[1,3,§], Alessandro A. Scandurra[1,4], Stefan Burén[1], Luis M. Rubio[1*], and Carlos Echavarri-Erasun[1*]

Author Affiliations

[1]Centro de Biotecnología y Genómica de Plantas, Universidad Politécnica de Madrid (UPM) - Instituto Nacional de Investigación y Tecnología Agraria y Alimentaria (INIA), Campus Montegancedo UPM, 28223-Pozuelo de Alarcón, Madrid, Spain.

Author Information Notes

*Corresponding authors: carlos.echavarri@upm.es; lm.rubio@upm.es

[§]These authors contributed equally

[2]Current address: Department of Chemistry, University of California, Davis, CA 95616, USA

[3]Current address: Department of Biochemistry, Virginia Tech, Blacksburg, VA 24061, USA

[4]Current address: Merck, Cambridge Science Park, Cambridgeshire, CB4 0WE, UK

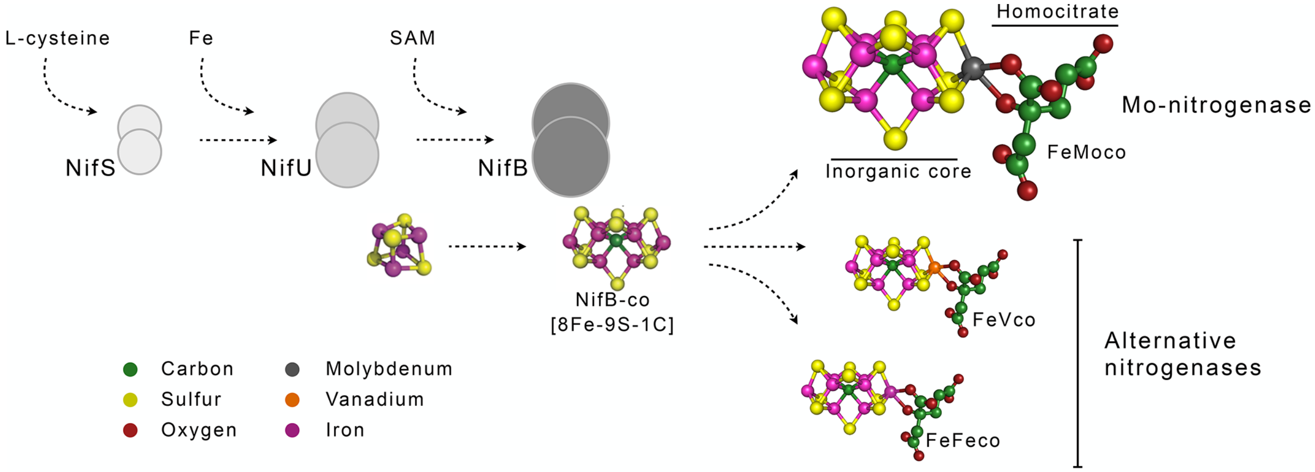

Supplementary Figure 1. Role of NifB in nitrogenase cofactor biosynthesis. NifB uses two [4Fe-4S] cluster units and SAM as substrates to synthesize a diamagnetic [8Fe-9S-C] cluster that serves as biosynthetic precursor to the active-site cofactors of all known nitrogenases. FeMo-co: iron-molybdenum cofactor; FeV-co: iron-vanadium cofactor; FeFe-co: iron-only cofactor.

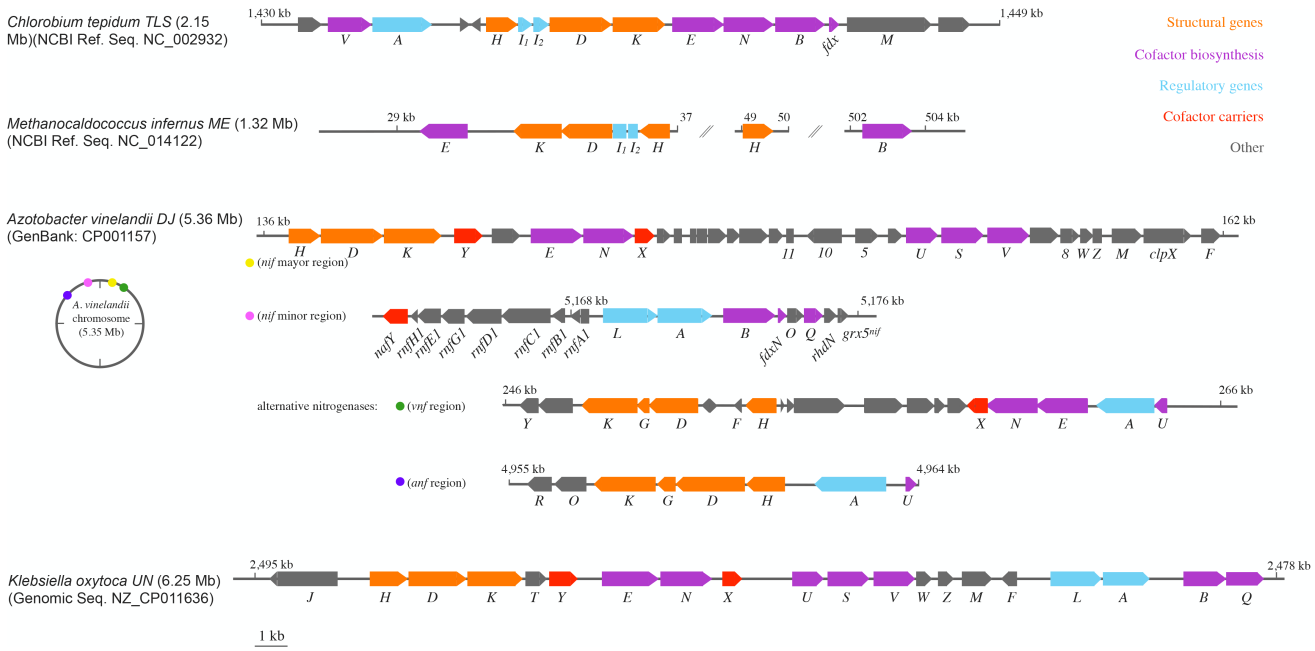

Supplementary Figure 2. Schematics of *nif* gene clusters in *C. tepidum*, *M. infernus*, *A. vinelandii* and *K. oxytoca*. *C. tepidum* and *K. oxytoca* carry single 12-kb and 20-kb *nif* genes clusters, respectively. *M. infernus* carries a *nifHI$_1$I$_2$DKE* gene cluster and separate copies of *nifH* and *nifB*. *A. vinelandii* has multiple nitrogen fixation clusters including *nif* for the Mo-nitrogenase, and *vnf* and *anf* regions for the alternative nitrogenases.

Biological information regarding *M. infernus* and *C. tepidum* strains:

M. infernus was first described as a hyperthermophilic lithotrophic methanogen isolated from deep-sea hydrothermal vents located at 3000 m depth in the Mid-Atlantic ridge. *M. infernus* presents a single chromosome of about 1.33 Mb predicted to encode for 1485 proteins. Although genome analysis reveals a basic *nif* configuration that could – theoretically – assemble a functional nitrogenase no study has yet been conducted that demonstrates *M. infernus* growth under diazotrophic conditions. Biochemical complementation assays proved that NifB$_{Mi}$ expressed and purified from *E. coli* was able to support FeMo-co biosynthesis *in vitro* when complemented with other purified Nif proteins.

C. tepidum TLS is a green-sulfur bacterium within the Chlorobia phylum that possesses a single 2.154 Mb chromosome. This bacterium performs anoxygenic photosynthesis and N_2 fixation when conditions are adequate. It was originally isolated from hot springs rich in sulfide in New Zeeland.

To date, *C. tepidum* is the only thermophilic species in the Chlorobium genus with an optimum growth temperature of 48ºC. *C. tepidum nif* genes are concentrated in a single operon and most genes appear related to archaea homologs, suggesting that nitrogen fixation in *C. tepidum* may have occurred by lateral gene transfer.

	Methanocaldococcus infernus	***Chlorobium tepidum***
Domain	archaea	bacteria
	methanogens	chlorobiae
NCBI TAXID	573063	194439
NCBI Genome	NC_014122.1	NC_002932.3
Genome size (Mb)	1.33	2.19
Predicted proteins	1485	1515
References (main text)	Jeanthon et al., 1998	Eisen et al., 2002

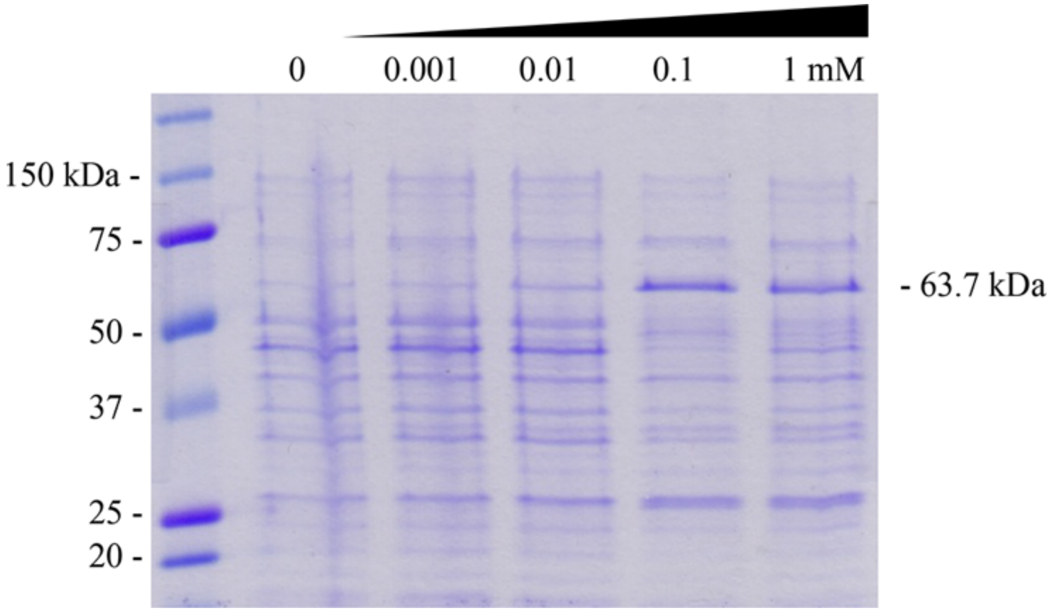

Supplementary Figure 3. Expression of NifB$_{Ko}$-ΔC in *K. oxytoca*. IPTG titration to test expression of GST-NifB$_{Ko}$-ΔC in *K. oxytoca*. Cells from IPTG induced cultures were collected and boiled in Laemmli buffer. Whole cell proteins were separated by SDS-PAGE and stained with Coomassie blue. Lane 1, molecular weight markers; lane 2, whole-cell protein profile in the absence of IPTG; lanes 3-6, whole-cell protein profiles 4 h after IPTG induction (1 μM uo to 1 mM IPTG). The 63.7 kDa protein corresponds to the size of GST-NifB$_{Ko}$-ΔC.
